# Is sitagliptin effective for SARS-CoV-2 infection: false or true prophecy?

**DOI:** 10.1007/s10787-022-01078-9

**Published:** 2022-09-30

**Authors:** Basil Mohammed Alomair, Hayder M. Al-kuraishy, Ali K. Al-Buhadily, Ali I. Al-Gareeb, Michel De Waard, Engy Elekhnawy, Gaber El-Saber Batiha

**Affiliations:** 1 Internal Medicine, Endocrinology and Diabetes Department of Medicine, College of Medicine, Aljouf University, Aljouf, Saudi Arabia; 2 Department of Pharmacology, Toxicology and Medicine, College of Medicine, Al-Mustansiriyah University, Baghdad, 14132 Iraq; 3 Department of Clinical Pharmacology, Medicine and Therapeutic, Medical Faculty, College of Medicine, Al-Mustansiriyah University, Baghdad, 14132 Iraq; 4 Smartox Biotechnology, 6 rue des Platanes, 38120 Saint-Egrève, France; 5 l’Institut du Thorax, Inserm UMR 1087/CNRS UMR 6291, Nantes, France; 6 Université de Nice Sophia-Antipolis, LabEx, Ion Channels, Science and Therapeutics, Valbonne, France; 7 Pharmaceutical Microbiology Department, Faculty of Pharmacy, Tanta University, Tanta, 31527 Egypt; 8 Department of Pharmacology and Therapeutics, Faculty of Veterinary Medicine, Damanhour University, Damanhour, 22511 AlBeheira Egypt

**Keywords:** Coronavirus disease 2019, Dipeptidyl peptidase-4, Sitagliptin, Inflammatory signaling pathways

## Abstract

Coronavirus disease 2019 (Covid-19) is caused by severe acute respiratory syndrome type 2 (SARS-CoV-2). Covid-19 is characterized by hyperinflammation, oxidative stress, and multi-organ injury (MOI) such as acute lung injury (ALI) and acute respiratory distress syndrome (ARDS). Covid-19 is mainly presented with respiratory manifestations; however, extra-pulmonary manifestations may also occur. Extra-pulmonary manifestations of Covid-19 are numerous including: neurological, cardiovascular, renal, endocrine, and hematological complications. Notably, a cluster of differentiation 26 (CD26) or dipeptidyl peptidase-4 (DPP-4) emerged as a new receptor for entry of SARS-CoV-2. Therefore, DPP-4 inhibitors like sitagliptin could be effective in treating Covid-19. Hence, we aimed in the present critical review to assess the potential role of sitagliptin in Covid-19. DPP-4 inhibitors are effective against the increased severity of SARS-CoV-2 infections. Moreover, DPP-4 inhibitors inhibit the interaction between DPP-4 and scaffolding proteins which are essential for endosome formation and replication of SARS-CoV-2. Therefore, sitagliptin through attenuation of the inflammatory signaling pathway and augmentation of stromal-derived factor-1 (SDF-1) may decrease the pathogenesis of SARS-CoV-2 infection and could be a possible therapeutic modality in treating Covid-19 patients. In conclusion, the DPP-4 receptor is regarded as a potential receptor for the binding and entry of SARS-CoV-2. Inhibition of these receptors by the DPP-4 inhibitor, sitagliptin, can reduce the pathogenesis of the infection caused by SARS-CoV-2 and their associated activation of the inflammatory signaling pathways.

## Introduction

Coronavirus disease 2019 (Covid-19) is principally triggered by various variants of severe acute respiratory syndrome type 2 (SARS-CoV-2) including α, β, and omicron variants (Al-Kuraishy et al. 2021a; Schmidt et al. 2022). Covid-19 is characterized by hyperinflammation, oxidative stress, and multi-organ injury (MOI) such as acute lung injury (ALI) and acute respiratory distress syndrome (ARDS) (Al-Kuraishy et al. 2021b; Elekhnawy and Negm 2022). Covid-19 patients are frequently asymptomatic in most of the cases (nearly up to 85%). Nevertheless, 15% of Covid-19 patients may present with moderate to severe form due to the advancement of SARS-CoV-2 infection with the propagation of ALI. In addition, 5% of Covid-19 patients may develop a serious form due to the development of ARDS that requires ventilator support and mechanical ventilation (Al-kuraishy et al. 2022c; Attallah et al. 2021). Covid-19 is particularly presented with respiratory manifestations; however, extra-pulmonary manifestations may propagate a critical form of Covid-19. Extra-pulmonary manifestations of Covid-19 are numerous including: neurological, cardiovascular, renal, endocrine, and hematological complications (Onohuean et al. 2021; Al-Kuraishy et al. 2021c). Of interest, SARS-CoV-2 uses angiotensin-converting enzyme 2 (ACE2) as a receptor and entry-point in the host cells (Al-Kuraishy et al. 2021c). Down-regulation of ACE2 by SARS-CoV-2 induces dysregulation of the renin–angiotensin system with augmentation of vasoconstrictor angiotensin II (AngII) and reduction of vasodilator of Ang1-7 (Schmidt et al. 2022). These changes together with the oxidative and inflammatory disorders can cause endothelial dysfunction, coagulation disorders, and the development of critical ALI/ARDS (Al-Kuraishy et al. 2021b).

Notably, the cluster of differentiation 26 (CD26) or dipeptidyl peptidase-4 (DPP-4) emerged as a new receptor for entry of SARS-CoV-2 (Al-Kuraishy et al. 2021e). Therefore, DPP-4 inhibitors like sitagliptin could be effective in treating Covid-19. Thus, the main aim of the present critical review was to assess the potential role of sitagliptin in Covid-19.

## DPP-4 inhibitors and viral infections

**Fig. 1:**
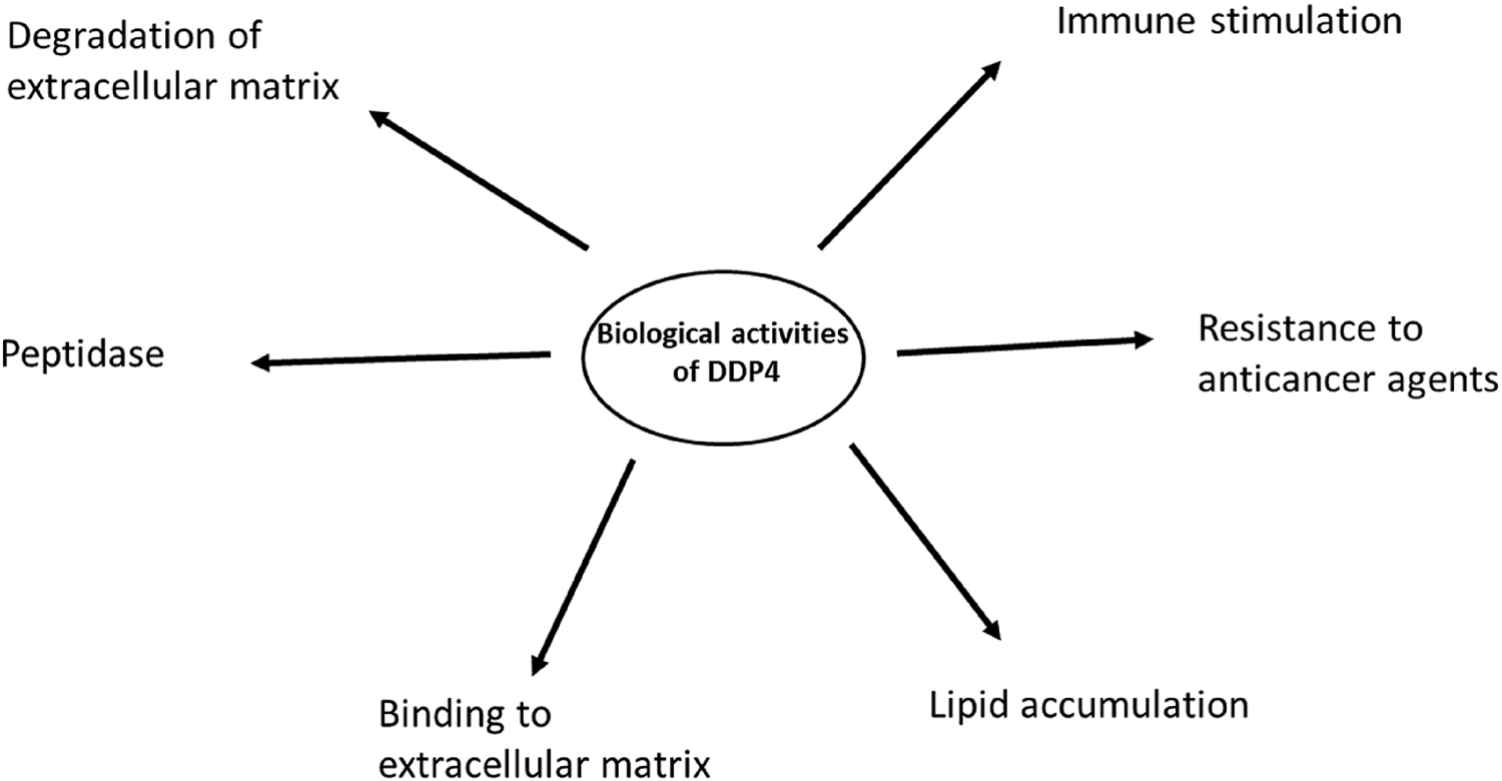
Various biological activities of DPP-4

DPP-4 is highly expressed in different immune cells and they are involved mainly in the regulation of blood glucose and inflammation. DPP-4 induces cleavage of glucagon-like peptide (GLP-1) and other incretins (Al-Kuraishy et al. 2020). DPP-4 inhibitors are widely used and tolerated in the management of type 2 diabetes mellitus (T2DM) with minimal or no hypoglycemia as an adverse effect (Al-Kuraishy et al. 2020). DPP-4 receptors are also involved in the regulation of other biological processes like immune response during viral infections owing to their expression on T cells, B cells, natural killer (NK) cells (Elekhnawy et al. 2021a), and macrophages (Amori et al. 2007) as shown in Fig. 1.

Previous meta-analyses illustrated that using of DPP-4 inhibitors was linked with a higher risk of respiratory viral infections (Amori et al. 2007). Similarly, a controlled study depended on the database of the World Health Organization (WHO) also implicates the use of DPP-4 inhibitors in increasing the risk of different viral infections (Yang et al. 2016). However, a recent meta-analysis by Yang et al. (2016) illustrated that uses of DPP-4 inhibitors were not related to increased risk of viral infections as compared with placebo. In addition, there is an updated meta-analysis which showed that that using DPP-4 inhibitors was not linked with a higher risk of respiratory viral infections (Grenet et al. 2021).

The immunological effects of DPP-4 inhibitors are related to the decrease of T cell activity and release of co-stimulatory chemokines and cytokines with upregulation of immunosuppressive cytokines (Yang et al. 2016; Grenet et al. 2021). Though these effects were not confirmed in experimental and preclinical studies.

These observations suggested that the uses of DPP-4 inhibitors in T2DM patients are safe and do not increase the risk of viral infections. In addition, immunomodulatory effects of DPP-4 inhibitors may reduce exaggeration of the immune response in viral infections; thereby decreasing the risk of immune-mediated tissue injury as in Covid-19 (Al-Kuraishy et al. 2021e).

## DPP-4 inhibitors and Covid-19

Many recent studies reported that DPP-4 inhibitors are effective in decreasing the severity of SARS-CoV-2 infections (Al-Kuraishy et al. 2021e; Noh et al. 2021). Of note, DPP-4 receptors are highly expressed in the case of obesity and T2DM (Grenet et al. 2021). Upregulation of DPP-4 receptors in the respiratory tract in obese and T2DM patients increase the risk for SARS-CoV-2 infection (Noh et al. 2021). Thus, overexpression of the membrane-bound DPP-4 increases the entry of SARS-CoV-2 into the host cells and is associated with augmentation of Covid-19 severity (Krejner-Bienias et al. 2021). However, soluble DPP-4 (sDPP-4) which are mainly released from the activated T lymphocytes may have a protective activity by neutralizing SARS-CoV-2 spike protein and preventing the binding with the bounded DPP-4 (Krejner-Bienias et al. 2021). It has been shown that sDPP-4 is decreased in SARS-CoV-2 infection and is correlated with lymphopenia in patients suffering from severe Covid-19 (Krejner-Bienias et al. 2021).

Moreover, DPP-4 inhibitors inhibit the interaction between DPP-4 and scaffolding proteins which are essential for endosome formation and replication of SARS-CoV-2 (Bardaweel et al. 2021). Furthermore, DPP-4 inhibitors have potent anti-inflammatory effects by reducing interleukin (IL-6) serum levels in Covid-19 patients. This property may diminish the risk of the progress of the cytokine storm (Bardaweel et al. 2021). Remarkably, an Italian prospective study comprising 338 Covid-19 patients revealed that the use of sitagliptin was correlated with a significant reduction in hospitalization time, severity, and mortality (Solerte et al. 2020). A narrative review and meta-analysis demonstrated inconsistent findings on the association between the use of DPP-4 inhibitors and clinical outcomes of Covid-19 patients (Bonora et al. 2021).

In this state, most of after mentioned studies did not discuss the possible beneficial mechanistic pathways of DPP-4 inhibitors in the amelioration of inflammatory signaling pathways in SARS-CoV-2 infection.

## Mechanisms of DPP-4 inhibitors in Covid-19

Several inflammatory signaling pathways are involved in the pathogenesis of the infections caused by SARS-CoV-2. The nod-like receptor pyrin 3 receptor (NLRP3) inflammasome is concerned with the activation of natural killer cells and the nuclear factor kappa β (NF-κβ) signaling pathway with the release of interferon gamma (INF-γ) (Al-Kuraishy et al. 2022a; Elekhnawy et al. 2021b). Suppression of NLRP3 inflammasome may decrease exaggerated immune response-induced organ injury (Al-Kuraishy et al. 2022a). As well, toll-like receptor (TLR4) is also activated in severe Covid-19 (Mostafa-Hedeab et al. 2022). Of interest, sitagliptin blocks activation of TLR4/NF-κβ/NLRP3 inflammasome axis in acute liver injury (El-Kashef and Serrya 2019).

Undoubtedly, p38 mitogen-activated protein kinase (p38MAPK) is a pro-inflammatory pathway connected with the progress of ALI and acute cardiac injury in Covid-19 (Al-Kuraishy et al. 2022b). Overactivation of p38MAPK in Covid-19 might be due to down-regulation of ACE2 and upsurge of AngII level. In addition, SARS-CoV-2 can in a straight line activate the p38MAPK signaling pathway causing endothelial dysfunction, vasoconstriction, and thrombosis (Gaohong et al. 2019). Gaohong et al. (2019) confirmed that sitagliptin inhibits p38MAPK signaling pathway-induced renal tubules epithelial cell injury in the experimental animals.

Likewise, the mechanistic target of the rapamycin (mTOR) pathway which is the intimate regulator of cell growth, proliferation, metabolism, and survival is found to be activated during SARS-CoV-2 infection. Moreover, it has a role in the transcription and mRNA translation of SARS-CoV-2 particles (Elekhnawy et al. 2022). Notably, sitagliptin attenuates cadmium-induced testicular injury by inhibiting the activation of the mTOR pathway in rats (Arab et al. 2021).

Further, stromal-derived factor-1 (SDF-1) which regulates immune response and migration of neutrophils is cleaved by DPP-4 (Mirabelli et al. 2020). Augmentation of SDF-1 by DPP-4 inhibitors may lead to a reduction in the severity of SARS-CoV-2 infection (Mirabelli et al. 2020). Besides, DPP-4 inhibitors increase the level of GLP-1 which by their anti-inflammatory and immunoregulatory properties may suppress SARS-CoV-2-induced hyperinflammation and immune dysregulation (Al-kuraishy et al. 2021d; Mirzaei et al. 2021).

**Fig. 2:**
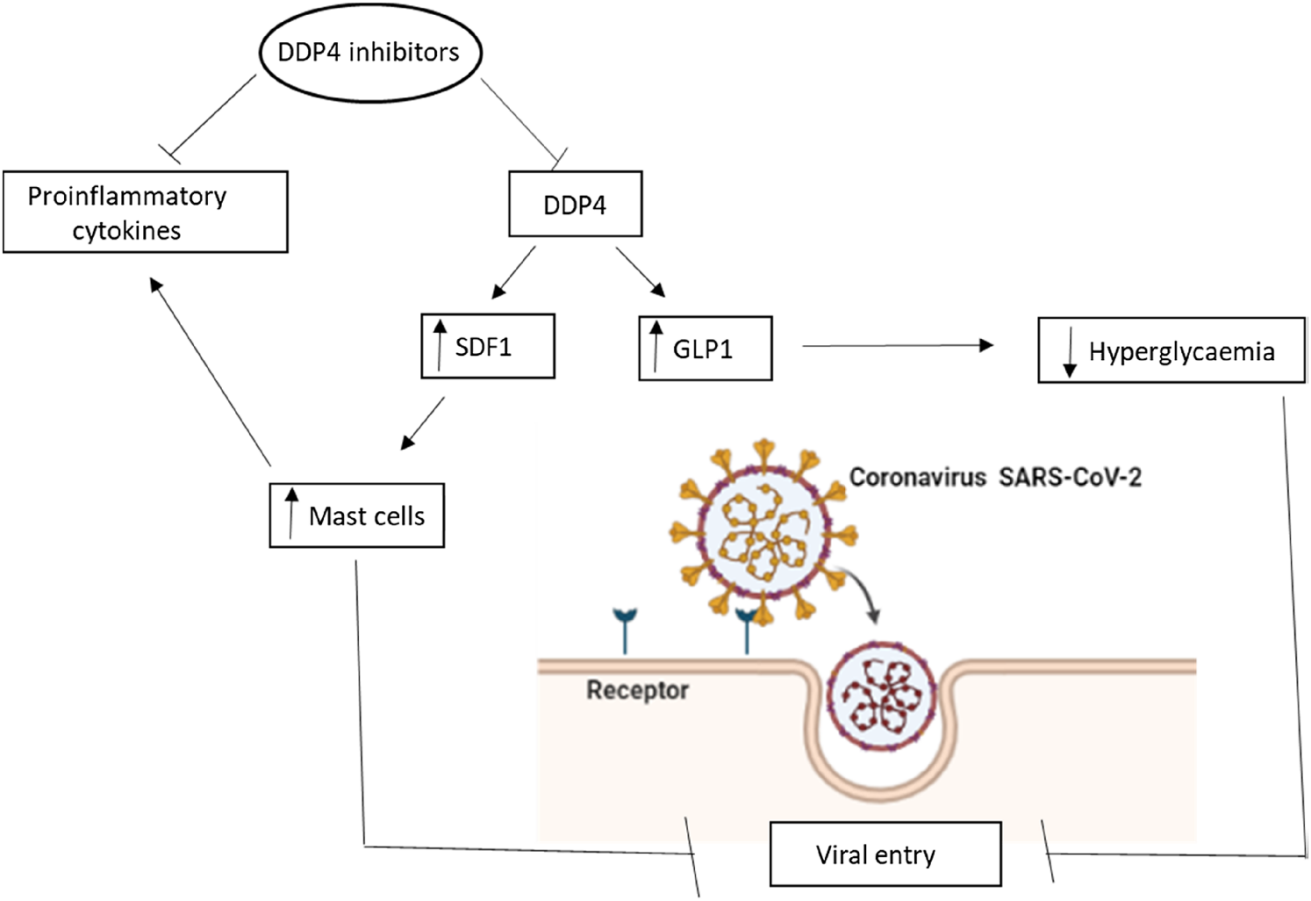
The potential role of DPP-4 inhibitors in Covid-19

Therefore, sitagliptin through attenuation of these inflammatory signaling pathways and augmentation of SDF-1 may decrease the pathogenesis of SARS-CoV-2 infection and could be a possible therapeutic modality in treating Covid-19 patients as shown in Fig. 2.

## Conclusions

DPP-4 receptors are highly expressed in T2DM and they are linked with the propagation of the inflammatory disorders. DPP-4 receptors are regarded as potential receptors for binding and entry of SARS-CoV-2. Inhibition of these receptors by the DPP-4 inhibitor, sitagliptin, can reduce the pathogenesis of SARS-CoV-2 infection and the associated activation of the inflammatory signaling pathways. This critical review excites for further clinical and preclinical studies to confirm the potential role of sitagliptin and other DPP-4 inhibitors in Covid-19.

## Data Availability

All data are available in the manuscript.
